# Pregnancy-care intentions and practice among family medicine physicians: residents, obstetric fellows, and fellowship alumni

**DOI:** 10.1093/fampra/cmaf006

**Published:** 2025-09-09

**Authors:** Robert William Owens, Mimi Dahlin, Emmanuel Adediran, Andrew Curtin, Saskia Spiess, Laura Elizabeth Moreno, Katherine T Fortenberry, Thomas Carlyle Whittaker, Dominik Ose

**Affiliations:** Department of Family and Preventative Medicine, Division of Family and Community Medicine, University of Utah, 310 Wakara Way, Salt Lake City, UT 84108, United States; School of Medicine, University of Utah, 30 N 1900 E, Salt Lake City, UT 84132, United States; Department of Family and Preventative Medicine, Division of Family and Community Medicine, University of Utah, 310 Wakara Way, Salt Lake City, UT 84108, United States; Department of Family and Preventative Medicine, Division of Family and Community Medicine, University of Utah, 310 Wakara Way, Salt Lake City, UT 84108, United States; Department of Family and Preventative Medicine, Division of Family and Community Medicine, University of Utah, 310 Wakara Way, Salt Lake City, UT 84108, United States; Department of Family and Preventative Medicine, Division of Family and Community Medicine, University of Utah, 310 Wakara Way, Salt Lake City, UT 84108, United States; Department of Family and Preventative Medicine, Division of Family and Community Medicine, University of Utah, 310 Wakara Way, Salt Lake City, UT 84108, United States; Department of Family and Preventative Medicine, Division of Family and Community Medicine, University of Utah, 310 Wakara Way, Salt Lake City, UT 84108, United States; Community Health Clinics, 610 S 200 E, Suite B, Salt Lake City, UT 84111, United States; Department of Family and Preventative Medicine, Division of Family and Community Medicine, University of Utah, 310 Wakara Way, Salt Lake City, UT 84108, United States; Faculty of Health and Healthcare Sciences, Westsächsische Hochschule Zwickau, Kornmarkt 1, 08056 Zwickau, 08012 Zwickau, Germany

**Keywords:** pregnancy, pregnancy outcome, health equity, education, medical, graduate, fellowships and scholarships, obstetrics

## Abstract

**Background:**

Pregnancy care in the USA is in crisis, particularly in rural areas. Shortages and maldistribution of care are contributing factors. Family medicine (FM) physicians could be crucial to addressing the crisis.

**Objectives:**

This study compared pregnancy and rural practice intentions of FM residents and FM obstetrics (FMOB) fellows, and current practices of FMOB fellowship alumni.

**Method:**

In this cross-sectional survey study, 25 FM residents, 7 FMOB fellows, and 37 FMOB fellowship alumni completed practice intentions or actual practice surveys. Variables of interest included intention or practice in rural locations and medically underserved areas, and pregnancy-care intentions or practice, including items on delivery types and advanced obstetrics. We used Fisher’s exact test to compare residents, fellows, and alumni.

**Results:**

Trainee intention and alumni practice were similar in practice characteristics except a higher rate of residents (80%) and fellows (100%) intended to practice in a medically underserved area (MUA) than alumni (29%) who practiced in an MUA (*P* < .001 and *P* = .001, respectively). Specific to pregnancy care, fellows and alumni respectively intended and provided low-risk, high-risk, and cesarean deliveries, and advanced obstetrics at higher rates than resident intentions.

**Discussion:**

Results suggest FMOB fellows are more likely intend to provide pregnancy-related care compared to FM residents, and alumni provide pregnancy-related care at rates similar to fellow intentions. Few FM residents complete obstetrics fellowships. FMOB fellowships alone cannot sufficiently address care shortages. Expanding and promoting FMOB fellowships would increase the pregnancy -care workforce, but more is needed for FM to realize its potential to resolve the crisis.

**Key messages**

- Pregnancy care in the USA is in crisis.
- Family medicine (FM) physicians can address pregnancy-care shortages.
- FM obstetrics (FMOB) fellows have greater intention to provide pregnancy care.
- FMOB alumni provide pregnancy care at higher rates.
- Expanding and promoting FM OB fellowships can address pregnancy-care shortages.

## Background

The USA has the highest rate of pregnancy-related mortality among industrialized nations [1]. Maternal mortality in the USA has increased steadily since 1987 and peaked sharply during the Corona Virus Disease 2019 (COVID-19) pandemic (32.9 deaths per 100 000 live births) [2]. Whereas the mortality rate in 2022 (22.3 deaths per 100 000 live births) decreased from the pandemic spike, it remains higher than the pre-pandemic rate, suggesting an ongoing increasing trend. Infant mortality is higher in the USA than other industrialized nations [3]. This pregnancy and birth care crisis is especially acute in rural areas. Remote and sparsely populated regions have the highest rates of maternal morbidity and mortality, and infant mortality [2, 4].

The crisis is complex and multicausal. The severe shortage of primary care physicians and particularly providers of pregnancy care are notable contributing factors. The Health Resources and Services Administration (HRSA) projects a shortage of 5170 obstetrician and gynecology physicians (OBGYNs) by 2030 [5]. In addition to OBGYNs, birthing people often rely on family medicine (FM) physicians for pregnancy care, especially in rural areas where the demand may be too low to sustain an OBGYN practice. However, FM participation in pregnancy care has decreased [6, 7], further contributing to care shortages.

In addition to provider shortages, the care available is inequitably distributed. For example, almost half of US counties do not have a practicing OBGYN [8], and over a third of counties in the USA are considered maternity care deserts (counties with no hospital or birth centers which provide obstetric care and limited or no obstetric providers). Additionally, maternity care deserts are increasing in rural areas [9].

The potential of FM providers to play an important role in addressing the crisis is well established [10–12]. The number, distribution, and medical training of FM physicians uniquely suit them to address the pregnancy and birth care crisis in the USA [10]. FM physicians make up the largest share of the primary care physician workforce [13]. Whereas OBGYN and maternal-fetal medicine specialists tend to be concentrated in urban areas [14], FM physicians are distributed similarly to the general population, making them an important staple for pregnancy care in rural and underserved areas. Furthermore, FM physicians are trained to care for patients of all genders, ages, and backgrounds [15], qualifying them to provide continuity care including pre-conception counseling, prenatal care, labor and delivery, postpartum care, transgender care, preventive, and chronic disease management for the birthing patient and pediatric care for the infant.

Although FM physicians are well-suited to play a role in addressing pregnancy-care shortages, they face barriers and challenges to providing pregnancy care. For example, residency training in pregnancy care varies considerably across programs and in general is not sufficient to prepare residents to provide full-spectrum pregnancy care independently [12]. Recent changes to residency training requirements, including an optional training track emphasizing comprehensive pregnancy care, have the potential to increase resident preparation [10]. Additionally, residencies may provide innovative curriculum around obstetrics resulting in greater preparedness and increased provision of pregnancy care after residency [16].

In addition to enhancing and improving residency training, family medicine obstetrics (FMOB) fellowships are integral to preparing FM physicians to provide pregnancy care. FMOB fellowships offer additional training in obstetrical care post-residency. Fellowships provide advanced training in pregnancy care including high-risk prenatal care, outpatient procedural training, and c-section training [17]. What is more, residents who complete FMOB fellowships are more likely to provide pregnancy care than other FM physicians, and are likely to practice in rural and underserved areas where the need is greatest [17, 18].

Given the critical role FM physicians could play in addressing the pregnancy and birth care crisis, and the disproportionate burden of the crisis in rural areas, we surveyed FM residents and FMOB fellows regarding their intentions to provide pregnancy care and to practice in rural areas. We also surveyed alumni of an FMOB fellowship regarding their provision of pregnancy care and whether they practice in rural areas. This study seeks to explore the potential role FM could play in addressing the critical shortage of pregnancy-care providers in rural areas; specifically, we examine differences between the practice intentions of FM residents and FMOB fellows and compare the practice intentions of those groups to the actual practice of FMOB fellowship alumni.

## Method

### Study design, funding, and ethics statement

This cross-sectional study compared the practice intentions of FM residents and FMOB fellows and the current professional practices of FMOB fellowship alumni. We used surveys to measure the practice intentions and actual practice characteristics of participants. This project was supported by HRSA of the US Department of Health and Human Services (HHS) as part of a funding award. The contents are those of the authors and do not necessarily represent the official views of, nor endorsement by HRSA, HHS, or the US government. The University of Utah IRB exempted this study (IRB 00159399).

### Sample

**Figure 1.**
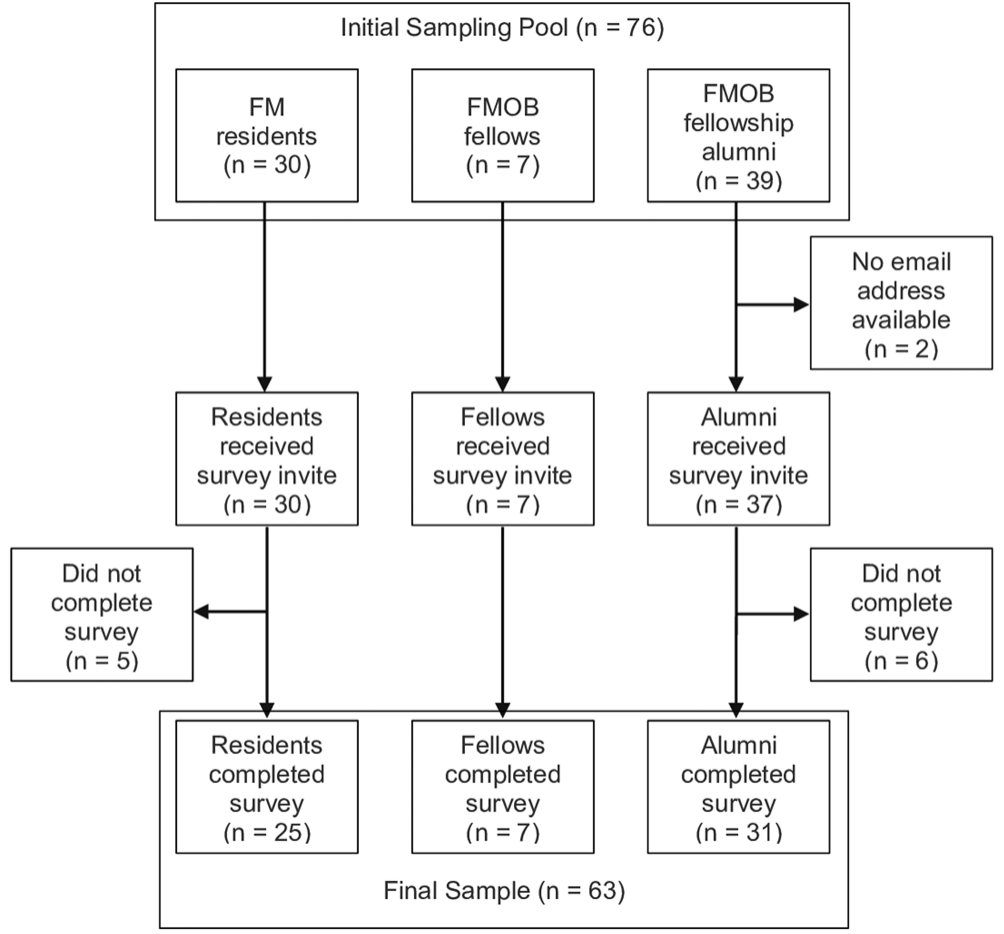
Flowchart of participants. Sampling pool, recruitment, and survey completion rates for residents, fellows, and fellowship alumni.

This study took place in Salt Lake City, Utah at the University of Utah (U of U). The sampling pool consisted of 30 FM residents, 7 FMOB fellows, and 39 fellowship alumni. FM residents were in their 1st, 2nd, or 3rd year of the U of U FM residency during the academic year 2022–2023. FMOB fellows were enrolled in the one-year fellowship during the academic year 2022–2023 (3 fellows) or 2023–2024 (4 fellows). Of the 39 alumni who had completed the FMOB fellowship between 2000 and 2022, we had current email addresses for and sent survey invitations to 37. In total, 25 (83%) FM residents, 7 (100%) FMOB fellows, and 31 (79%) fellowship alumni completed surveys (Fig. 1).

Demographic information (Table 1) indicates the sample was mostly female for FM residents, FMOB fellows, and fellowship alumni, 14 (56%), 7 (100%), and 17 (57%) respectively. The majority of all participants identified as White (FM residents 76%, *n* = 19; FMOB fellows 71%, *n* = 5; and fellowship alumni 88%, *n* = 13). Information about race was only available for 16 (55%) fellowship alumni.

**Table 1.** Demographic characteristics of residents, fellows, and fellowship alumni.

Demographic characteristic	Residents *n* (%)	Fellows *n* (%)	Alumni *n* (%)	Fisher’s Exact *P*		
				Res-Fel	Fel-Alum	Res-Alum
Gender						
Female	14 (56)	7 (100)	17 (57)	.066	.038	1
Male	11 (44)	0 (0)	12 (40)	.066	.072	.790
Prefer not to say	0 (0)	0 (0)	1 (3)	1	1	1
Race^*^						
White or Caucasian	19 (76)	5 (71)	13 (88)	1	.621	.494
Black or African American	1 (4)	1 (14)	1(6)	.374	.526	1
American Indian/Alaska Native	1 (4)	0 (0)	0 (0)	1	1	1
Asian	3 (12)	0 (0)	1 (6)	1	1	1
Another race	1 (4)	1 (14)	1 (6)	.374	.526	1
Prefer not to say	2 (8)	0 (0)	0 (0)	1	1	.522
Ethnicity^*^				1	1	.088
Non-Hispanic	22 (88)	7 (100)	16 (100)			
Hispanic	3 (12)	0 (0)	0 (0)			

^*^Race and ethnicity information available for *n* = 16 of alumni. Some residents reported multiple races; percentages exceed 100.

### Survey development

The study utilized two surveys, one to measure practice intentions in FM residents and FMOB fellows and one to measure aspects of practice in fellowship alumni. After a review of literature regarding FM residents intentions to provide pregnancy care and practice in rural areas, we developed the intentions survey. We created the survey using the Qualtrics XM online survey platform. FM physician faculty of the residency and fellowship reviewed and piloted the survey. We revised survey items based on feedback. We developed the fellowship alumni survey to reflect content of the intentions survey. Three alumni of the FMOB fellowship program reviewed and piloted the fellowship alumni survey, and we revised it based on their feedback. Alumni who reviewed the survey were also participants who completed the survey.

The intentions survey asked FM residents and FMOB fellows whether they intended to practice in rural and medically underserved areas (MUAs), and whether they intended to provide pregnancy care. Additional items asked about their intentions to provide pregnancy-care services such as prenatal care, low-risk deliveries, high-risk deliveries, cesarean deliveries, and advanced or high-risk obstetric services. The response scale for practice intentions included “definitely not,” “probably not,” “maybe,” “probably,” and “definitely.”

The fellowship alumni survey included items regarding the current practice of fellowship alumni. Specifically, we asked whether they: practice in a rural or frontier area, practice in an MUA, and whether they provide pregnancy care. The survey also asked fellowship alumni whether they provide prenatal care, and perform low-risk deliveries, high-risk deliveries, cesarean deliveries, and advanced or high-risk obstetrics.

### Data collection

All participants completed surveys via Qualtrics. FM residents and the first cohort of FMOB fellows received an email invitation to complete the survey in January 2023 and follow-up emails every 2 weeks through the end of March. We followed the same process for the second cohort of FMOB fellows in July of 2023. We sent email invitations to fellowship alumni for whom we had current email addresses in January of 2023 and sent follow-up reminders to those who had not completed the survey until April 2023.

### Analyses

Using RStudio, we compared the frequency of FM residents and FMOB fellows who “maybe,” “probably,” or “definitely” intend to include certain characteristics in their practice and compared those results with the frequency of fellowship alumni who reported actually having those characteristics in their practice. Due to the small group size of FMOB fellows, we used Fisher’s exact test which is well-suited for comparing frequencies with a small group size [19]. We performed Fisher’s exact test comparing all three groups on the variables of interest and found significant differences for all variables. Next, we performed follow-up Fisher’s exact testing comparing dyads of subgroups for each variable (i.e. FM residents versus FMOB fellows, FMOB fellows versus fellowship alumni, and FM residents versus fellowship alumni).

## Results

Our analysis compared FM residents, FMOB fellows, and fellowship alumni in terms of practice intentions for FM residents and FMOB fellows and characteristics of practice for fellowship alumni. Practice location outcomes of interest included practice intention and actual practice in a rural or frontier location and in an MUA. Practice characteristic outcomes of interest were intention to provide and actual provision of pregnancy care, including the intention to perform low-risk, high-risk, and cesarean deliveries, as well as the inclusion of advanced obstetrics care.

We found few significant differences in general practice intentions and characteristics between the three groups. In total, 80% (*n* = 20) of FM residents and all 7 FMOB fellows intended to practice in an MUA, significantly more than 29% (*n* = 9) of fellowship alumni who practiced in MUAs (FM resident-fellowship alumni *P* = 0.001, FMOB fellowship-fellowship alumni *P* < .001). We found no significant differences between the three groups in practice intention and actual practice in rural or frontier areas and intent to provide and actual provision of general pregnancy care (Table 2).

**Table 2.** General practice intentions/characteristics for residents, fellows, and fellowship alumni.

Practice characteristic	Residents *n* (%)	Fellows *n* (%)	Alumni *n* (%)	Fisher’s Exact *P*		
				Res-Fel	Fel-Alum	Res-Alum
Rural or frontier	7 (27)	5 (71)	9 (29)	.071	.077	1
Medically underserved area	20 (77)	7 (100)	9 (29)	.301	.001	<.001
Medical education	23 (88)	6 (86)	25 (81)	1	1	.488
Pregnancy-care provision	16 (62)	7 (100)	24 (77)	.073	.309	.246

We examined specific pregnancy-care practices and found FM residents had lower intentions to provide most kinds of pregnancy care compared to fellow intentions and fellowship alumni practice. For all delivery types, all FMOB fellows intended to provide, and more fellowship alumni provided services than FM residents who intended to provide low-risk (FM resident-fellowship alumni *P* = .017, FM resident-FMOB fellow *P* = 0.003), high-risk (FM resident-fellowship alumni *P *< .001, FM resident-FMOB fellow *P* < .001), and cesarean (FM resident-fellowship alumni *P* < .001, FM resident-FMOB fellow *P* < .001) deliveries. Intention and provision of advanced obstetrics were also higher for FMOB fellows and fellowship alumni compared to FM residents (FM resident-fellowship alumni *P* < .001, FM resident-FMOB fellow *P* < .001). For specific pregnancy services, there were no significant differences between fellow practice intentions and fellowship alumni actual practice. Finally, there were no significant differences between all three groups in practice intentions and actual provision of prenatal care (Table 3).

**Table 3.** Pregnancy-care intentions/practice for residents, fellows, and fellowship alumni.

Pregnancy-care practice detail	Residents *n* (%)	Fellows *n* (%)	Alumni *n* (%)	Fisher’s Exact *P*		
				Res-Fel	Fel-Alum	Res-Alum
Prenatal	16 (62)	7 (100)	23 (74)	.073	.307	.394
Low-risk deliveries	9 (35)	7 (100)	21 (68)	.003	.156	.017
High-risk deliveries	3 (12)	7 (100)	23 (74)	<.001	.307	<.001
Caesarean deliveries	3 (12)	7 (100)	20 (65)	<.001	.084	<.001
Advanced obstetrics	6 (23)	7 (100)	23 (74)	<.001	.307	<.001

## Discussion

The aim of this study was to understand the differences between pregnancy-care practice intentions of FM residents and FMOB fellows, and to compare the practice intentions of FM residents and FMOB fellows to the actual practice of FMOB fellowship alumni. Survey results suggest that for general practice characteristics, resident, and fellow intentions do not differ from actual fellowship alumni practice, except that trainees intend to practice in an MUA at a higher rate than fellowship alumni practice in an MUA. Looking at more specific aspects of pregnancy care, we found that FMOB fellows intentions align with fellowship alumni practice. Compared to FM residents, more FMOB fellows intended to provide, and more fellowship alumni did provide low-risk, high-risk, and cesarean deliveries, and advanced obstetric services. This finding is unsurprising given the demonstrated interest of FMOB fellows and fellowship alumni in pregnancy care.

Our findings regarding fellow pregnancy-care practice intentions and actual pregnancy care of fellowship alumni are encouraging given the pregnancy and birth care crisis and the service needs in rural areas. A high percentage of FMOB fellows in our sample intend to practice in rural areas, and all intend to provide pregnancy care including deliveries and advanced obstetrics. The alignment of fellow intentions with fellowship alumni practice suggests FMOB fellows are likely to realize their practice intentions.

In contrast, results pertaining to FM residents, although not surprising, are less encouraging given the pregnancy and birth care crisis. Although more than half of FM residents had some intention to provide pregnancy care, most of them intended to solely provide prenatal care. Just over a third of FM residents intended to provide low-risk deliveries; however, previous studies suggest fewer than half of FM residents who intend to provide prenatal care or perform deliveries do so in practice [6, 20, 21].

Although FM has the potential to play an important role in addressing the pregnancy and birth care crisis and is particularly well-suited to address service needs in rural areas, that potential is yet to be realized. Our results suggest FM intentions to provide pregnancy-related care are limited, which is consistent with previous research [6, 7] indicating FM participation in pregnancy care is decreasing. Any enthusiasm regarding the potential participation of FMOB fellows in addressing pregnancy-care gaps is tamped down by low numbers of FM residents who complete OB fellowships. Data collected on the resident certification examination application found that only 17% of completing FM residents intended to enroll in a fellowship. Of those who intended to enroll in a fellowship, just less than 13% (about 2% overall) intended to enroll in an OB fellowship [22]. This trend is particularly concerning given the evidence that FMOB fellowship-trained physicians provide quality of care comparable to that provided by OBGYN physicians [23] while also being able to care for the entire community in healthcare deserts.

### Strengths and limitations

This study has strengths and limitations. One strength of the study is the inclusion of multiple perspectives. The study provides a more comprehensive picture of the role of FM in addressing the pregnancy and birth care crisis by including FM residents, FMOB fellows, and fellowship alumni in the sample. There are some limitations associated with the sample, including low sample size and homogeneity. All participants were enrolled or completed programs in a single department at one university, limiting generalizability. Additionally, the inclusion of FM resident-fellowship alumni who had not completed an FMOB fellowship would have provided an even more complete picture. Another limitation is the use of cross-sectional data; whereas we draw comparisons between fellowship alumni practices and fellow intentions that assume similar trajectories for the two groups, cross-sectional analyses cannot confirm that FMOB fellows will realize their intentions to provide pregnancy care in rural areas based on the practices of fellowship alumni. Future studies utilizing longitudinal data could provide additional clarity. Finally, our data are based on self-report surveys, which may introduce recall or respondent bias. Particularly for measuring current practices of fellowship alumni, other data sources such as billing or electronic health records could increase validity.

## Conclusion

Pregnancy and birth care in the USA is in crisis, with rural areas bearing a disproportional burden of poor outcomes. Shortages of primary care providers for pregnancy care and maldistribution of care contribute to the crisis. FM physicians are uniquely suited to address care shortages. Unfortunately, FM participation in pregnancy care is dwindling. Our results suggest that FMOB fellows are likely to provide pregnancy care in rural areas. Whereas expanding and promoting FMOB fellowships may marginally increase the pregnancy-care workforce, especially in rural and underserved areas, alternative approaches are necessary. Increasing FM residency pregnancy-care training and exposure, incentivizing FM residents to pursue this specialty, and policy changes incentivizing FM participation are crucial steps. Only through a multi-pronged approach can FM realize its full potential in mitigating the US pregnancy and birth care crisis [10].

## Data Availability

Supplementary data are available on reasonable request to the corresponding author.
